# Electrochemiluminescence immunosensor for cytokeratin fragment antigen 21-1 detection using electrochemically mediated atom transfer radical polymerization

**DOI:** 10.1007/s00604-020-04677-x

**Published:** 2021-03-08

**Authors:** Lihe Jian, Xiaolan Wang, Lulu Hao, Yanju Liu, Huaixia Yang, Xiaoke Zheng, Weisheng Feng

**Affiliations:** 1 Pharmacy College, Henan University of Chinese Medicine, Zhengzhou, 450046 People’s Republic of China; 2 The Engineering and Technology Center for Chinese Medicine Development of Henan Province, Zhengzhou, 450046 People’s Republic of China

**Keywords:** Biosensor, Immunosensor, CYFRA 21-1, Electrochemiluminescence, eATRP

## Abstract

The cytokeratin fragment antigen 21-1 (CYFRA 21-1) protein is a critical tumor biomarker tightly related to non-small cell lung cancer (NSCLC). Herein, we prepared an effective electrochemiluminescence (ECL) immunosensor for CYFRA 21-1 detection using electrochemically mediated atom transfer radical polymerization (eATRP). The CYFRA 21-1 antigen was fixed on the electrode surface by constructing a sandwich type antibody-antigen-antibody immune system. The sensitivity of ECL was improved by using the eATRP reaction. In this method, eATRP was applied to CYFRA 21-1 detection antibody with N-acryloyloxysuccinimide as functional monomer. This is the first time that ECL and eATRP signal amplification technology had been combined. Under the optimized testing conditions, the immunosensor showed a good linear relation in the range from 1 fg mL^−1^ to 1 μg mL^−1^ at a limit of detection of 0.8 fg mL^−1^ (equivalent to ~ 134 molecules in a 10 μL sample). The ECL immunosensing system based on eATRP signal amplification technology provided a new way for rapid diagnosis of lung cancer by detecting CYFRA 21-1.

**Supplementary Information:**

The online version contains supplementary material available at 10.1007/s00604-020-04677-x.

**Figure:**
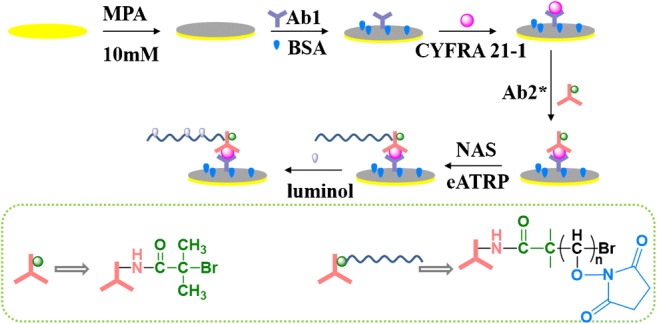
The paper prepared an electrochemiluminescence biosensor for ultrasensitive detection of CYFRA 21-1 via eATRP signal amplification strategy, which had the advantages of high sensitivity, reproducibility, and held potential prospect for analysis of low-abundance.

## Introduction

Lung cancer, typified by the growth of malignancy, is a grave threat to human life. The incidence and mortality ratio of lung cancer is rising rapidly. In general, lung cancer mainly includes two subtypes, namely, the small cell lung cancer and non-small cell lung cancer (NSCLC) [1]. Cytokeratin fragment antigen 21-1 (CYFRA21-1), located in the 36-kDa fragment of cytokeratin 19, is the most specific tumor marker of NSCLC [2]. Currently, an ultrasensitive determination of tumor biomarkers is critical for early screening and diagnosis of carcinoma patients [3, 4]. Many researches have shown that the CYFRA21-1 expression level in NSCLC sufferers is tightly bound to the disease screening and prognosis [5].

In general, several techniques have been extensively applied for detecting tumor biomarkers, such as radioimmunoassay [6], chemiluminescence assay [7], enzyme-linked immunosorbent assay [8], metalloimmunoassay [9], electrophoretic immunoassay [10], and electrochemiluminescence (ECL) [11]. Among these, ECL is widely used because of its low cost, speediness, wide range of analytes, and low background signal. Lung cancer has no obvious early symptoms and is only diagnosed in the terminal stage [12]. However, traditional ECL methods limit the detection of low-abundance biomarkers. Hence, it is critical to establish a highly sensitive CYFRA 21-1 detection method, which is significant for the early screening and detection of NSCLC.

In order to enhance the sensitivity of ECL, various techniques have been proposed, such as cleavage reaction [13], enzyme catalysis [14], and long-chain polymers [15]. However, the instability of enzyme catalysis and limitations of cleavage reaction limit their application. The signal amplification technique based on polymers is a stable, strong-applicability, and low-cost method [16]. Currently, atom transfer radical polymerization (ATRP) is one of the most frequent and significant methods used for polymer synthesis. It not only has a wide range of monomers, such as (meth) acrylamide, (meth) acrylate, styrenes, and acrylonitrile, but can also control the polymer thickness. ATRP is effective because of its various monomers, mild reaction conditions, and high polymerization yield. It can be successfully applied to polymer growth by forming a dynamic balance between dormant species at high concentrations and the active species at low concentrations [17].

In the study of ATRP, copper and its compounds are generally used as catalysts. Because they are somewhat toxic, new catalysts with low toxicity and high efficiency must be found [18]. Therefore, activators were developed for regenerating electron-transfer ATRP (ARGET ATRP) by adding reducing reagents to trigger ATRP at low-toxicity catalyst concentrations [19]. However, the by-products of ARGET ATRP activator regeneration might be toxic, causing unnecessary changes in the natural color of the long-chain polymers [20]. To triumph over this challenge, Matyjaszewski et al. applied an electrochemical potential to reduce the number of deactivators, which can effectively prevent the formation of the reaction by-products [21, 22]. Latterly, electrochemically mediated atom transfer radical polymerization (eATRP) has been widely developed in various fields because of its advantages of precise molecular-weight control, narrow molecular-weight distribution, and clear structure [23]. We have proven that signal amplification based on eATRP is an economical and efficient method for DNA detection [24]. Therefore, this study, for the first time, uses an ECL immunosensor via eATRP to detect CYFRA 21-1.

In this experiment, the CYFRA 21-1 detection antibody (Ab2) was introduced to a gold-electrode surface through two immunoreactions. Then, at constant potential, amount of electroactive polymers is grafted in situ from the initiation sites of eATRP with N-acryloxysuccinimide (NAS) as the monomer, which significantly amplifies the ECL signal. After adding N-hydroxysuccinimide (NHS)/1-ethyl-3-(3-dimethyllaminopropyl) carbodiimide hydrochloride (EDC), the carboxyl group of NAS is activated into an NAS ester, which condenses with luminol. Subsequently, considerable luminol is introduced to the electrode surface. The sensitivity of the biosensor is significantly improved using the signal amplification strategy.

Based on the amplification of eATRP signal, a simple and ultrasensitive ECL sensing system was established to detect CYFRA21-1 in human serum. NAS was employed as monomer to eATRP reaction using constant potential. Furthermore, compared with other methods, our method does not involve polymerase, expensive instruments and temperature requirements. Therefore, this method has the advantages of low excitation potential, good selectivity, simplistic operation, high sensitivity, and environmental friendliness. eATRP can also be applied to other types of protein detection, and has great application potential in the biomedical field.

## Experimental

### Materials and apparatus

The information on materials and apparatus has been described in the Supplementary Materials.

### Synthesis of initiator coupled with polyclone CYFRA 21-1 antibody (Ab2*)

First, 10 mM of 2-bromoisobutyric acid (8.35 mg) was dissolved in 60% ethanol. To activate the functional groups of 2-bromoisobutyric acid, 500 μL of 10 mM 2-bromoisobutyric acid was added to 1 M NHS (500 μL) and 4 M EDC (500 μL) for 1 h, with gentle stirring, at room temperature [25]. Subsequently, the initiator 2-bromoisobutyric acid was added to 10 mg mL^−1^ Ab2 solution and agitated gently for 2 h at 25 °C [26], where the amide bond immobilized 2-bromoisobutyric acid.

### Preparation of the ECL biosensor

Before each experiment, in order to obtain a smooth surface, a bare gold electrode needed to be mechanically treated with 0.3 and 0.05 μm alumina slurries, and then ultrasonically cleaned with anhydrous ethanol and ultrapure water. Afterward, chemical cleaning was carried out in a newly prepared solution of piranha (30% H_2_O_2_ and 98% H_2_SO_4_, 1:3 in volume) for 15 min. It was then treated in 0.5 M H_2_SO_4_ via cyclic voltammetry (CV); the electrode potential was between − 0.2 and 1.5 V, and a scan rate of 0.1 V s^−1^ until obtaining a repeatable CV. Finally, the gold electrode was sonicated in ultrapure water and dried with N_2_.

The pretreated gold electrode was immersed in 10 mM MPA at 37 °C overnight, and then immersed in a 400-μL solution containing 0.4 M EDC and 0.1 M NHS and incubated for 30 min to activate the carboxylic group. Subsequently, 10 μL Ab1 (1 pg mL^−1^) was directly dropped onto the electrode surface (Ab1/Au), and then reacted for 30 min. To block the nonspecific binding sites, the resultant electrode was reacted with 10 μL BSA (1 wt%) for 30 min, and then, 10 μL CYFRA 21-1 was added onto the Ab1-modified substrate at 37 °C for 30 min to capture antigen in the first immunoreaction (CYFRA21-1/Ab1/Au). The obtained electrode (CYFRA 21-1/Ab1/Au) was then incubated with 10 μL BSA (1 wt%) for 30 min. After washing, 10 μL Ab2* was dropped onto the CYFRA 21-1/Ab1/Au surface at 37 °C for 30 min to introduce Ab2* through the second immunoreaction (Ab2*/CYFRA 21-1/Ab1/Au). We soaked the electrode in the eATRP mixture and electrochemically polymerized it with the i-t curve at a constant potential for 40 min, to introduce considerable NAS. It was then immersed into a luminol solution for 4 h at 37 °C to bring luminol onto the polymer. In the luminol solution, NAS was pre-conjugated with luminol through EDC/NHS to achieve the maximum loading capacity for immunoassay. Finally, the luminescent intensity was measured with ECL in 0.1 M PBS (pH = 7.5) containing 10 mM H_2_O_2_. At 50 mV s^−1^, the potential applied to the working electrode in the CV measurement ranged from − 0.5 to 1.5 V. The emission window was placed in front of the PMT biased at 1000 V.

## Results and discussion

### Detection mechanism of the ECL immunosensor for CYFRA 21-1

**Scheme 1:**
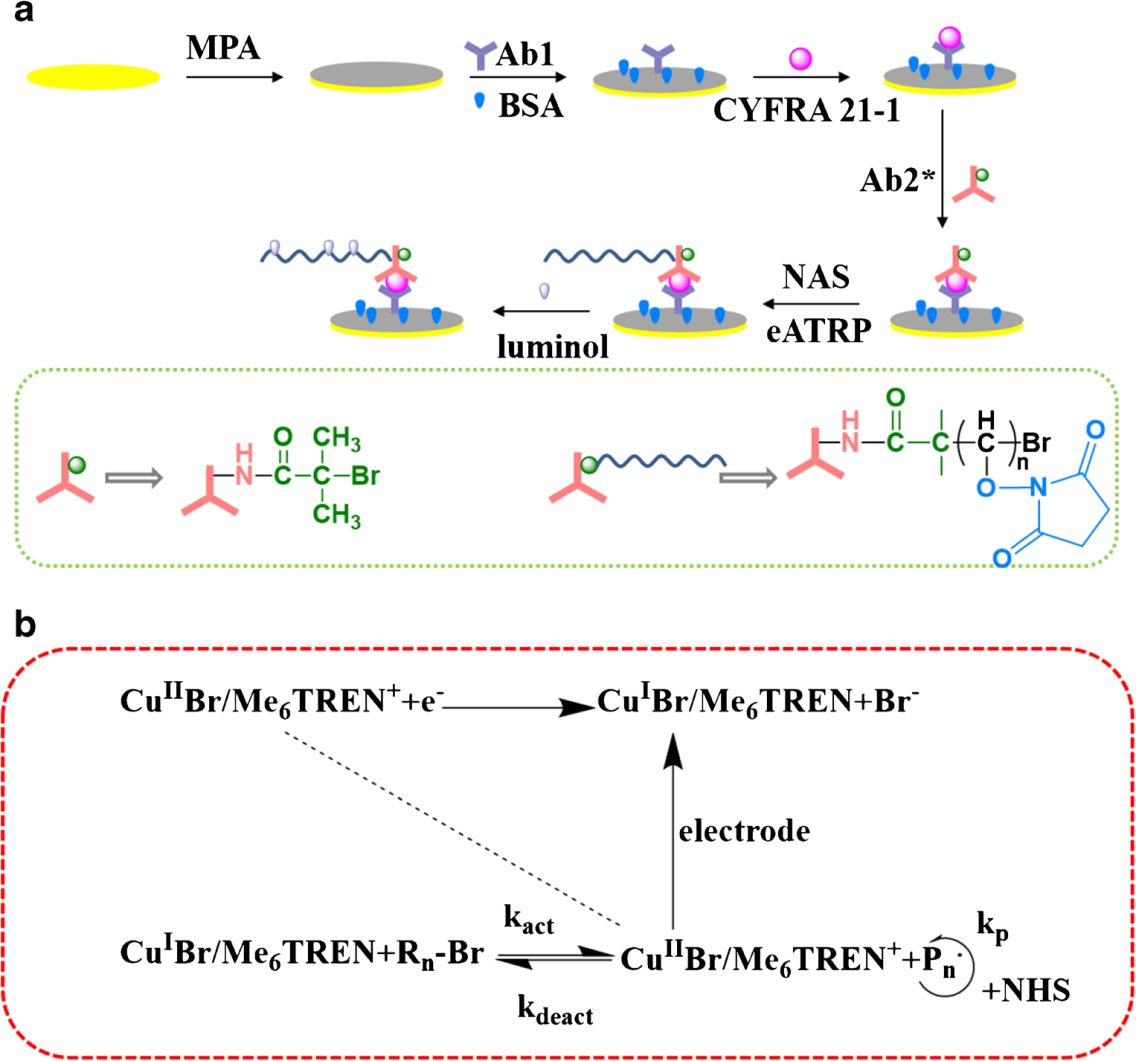
Schematic illustration of **a** the fabrication of the biosensor and **b** the mechanism of eATRP-based amplification strategy. The concentrations of CYFRA 21-1 were all 1 pg mL^−1^

The mechanism of the CYFRA 21-1 protein detection is presented in Scheme 1a. This biosensor is prepared using eATRP as the signal amplification method and luminol-H_2_O_2_ is the luminescent system. First, the self-assembly of the Au–S bond immobilizes the MPA on the electrode surface. Then, EDC/NHS activates the carboxyl group of MPA and the amide bond fixes the Ab1. After BSA blocks the residual binding sites, Ab2* is introduced into the gold-electrode surface through sandwich immunoassay. Because of the bromine group in Ab2*, it can provide recognition sites for eATRP, and numerous NASs are grafted on the electrode surface through eATRP.

After adding EDC/NHS, the carboxyl group of NAS is activated into an NAS ester that can react with luminol. Subsequently, considerable luminol is introduced to the electrode surface, and the ECL signal is amplified. In this study, an appropriate potential is applied to the working electrode to control the polymerization. Scheme 1 b depicts the principle of eATRP. In the eATRP, pH is an important factor affecting the stability of Cu^II^Br/Me_6_TREN and Cu^I^/Me_6_TREN complexes. Under acidic or alkaline conditions, it is not conducive to the participation of ligands in proton reactions. They are harmful to the growth of polymer chains. Therefore, the pH value of eATRP solution is 7.0. Initially, the Br-Cu^ǁ^/Me_6_TREN^+^ (catalytic complex) is readily backed to the Br-Cu^ǀ^/Me_6_TREN^+^ (active complex) through a constant potential, which can partially decompose into Br^−^ and Cu^ǀ^/Me_6_TREN^+^. Through the inner-sphere electron transfer, Cu^ǀ^/Me_6_TREN^+^ reacts with P_n_-Br (a dormant alkyl alide initiator) to Br-Cu^ǁ^/Me6TREN^+^ (generate deactivators) and spread P_n_ (free radicals), which are again involved in polymerization. The free radicals can be circulated by the P_n_P_m_ (terminator) and the monomer (NAS) or be inactivated back to the P_n_-Br (dormant species) through the Br-Cu^ǁ^/Me_6_TREN^+^ (high-oxidation-state catalyst) via a charge transfer on the outer sphere [27]. Then, Br-Cu^ǁ^/Me_6_TREN^+^ reacts with the P_n_P_m_ (coupled radicals) to form P_n_P_m_-X (long-chain polymers). Numerous NAS-labeled polymer chains grow continuously at each site by electrochemical-mediated polymerization, which significantly improves the detection sensitivity of CYFRA 21-1.

In the light of the literature, the possible equation for the reaction is summarized as follows [28–30]. Initially, there is a rapid generation of reactive species by H_2_O_2_ (Eq. 1), and simultaneously, luminol is electrooxidized to radical anions luminol^·−^ (Eq. 2). ${\mathrm{O}\mathrm{H}}^{\cdotp }/{\mathrm{O}}_2^{\cdotp -}$ functions as an oxidant and reacts with luminol^·−^, which is converted into an excited state (Eq. 3) and returns to the ground state by luminescence (Eq. 4).

1 $$ {\mathrm{H}}_2{\mathrm{O}}_2\to {\mathrm{O}\mathrm{H}}^{\cdotp }/{\mathrm{O}}_2^{\cdotp -} $$

2 $$ \mathrm{luminol}-{\mathrm{e}}^{-}-2{\mathrm{H}}^{+}\to {\mathrm{luminol}}^{\cdotp -} $$

3 $$ {\mathrm{luminol}}^{\cdotp -}+{\mathrm{O}\mathrm{H}}^{\cdotp }/{\mathrm{O}}_2^{\cdotp -}\to {\mathrm{luminol}}_{\mathrm{ox}}^{\ast } $$

4 $$ {\mathrm{luminol}}_{\mathrm{ox}}^{\ast}\to \mathrm{luminol}+\mathrm{hv} $$

### Characterization

**Fig. 1:**
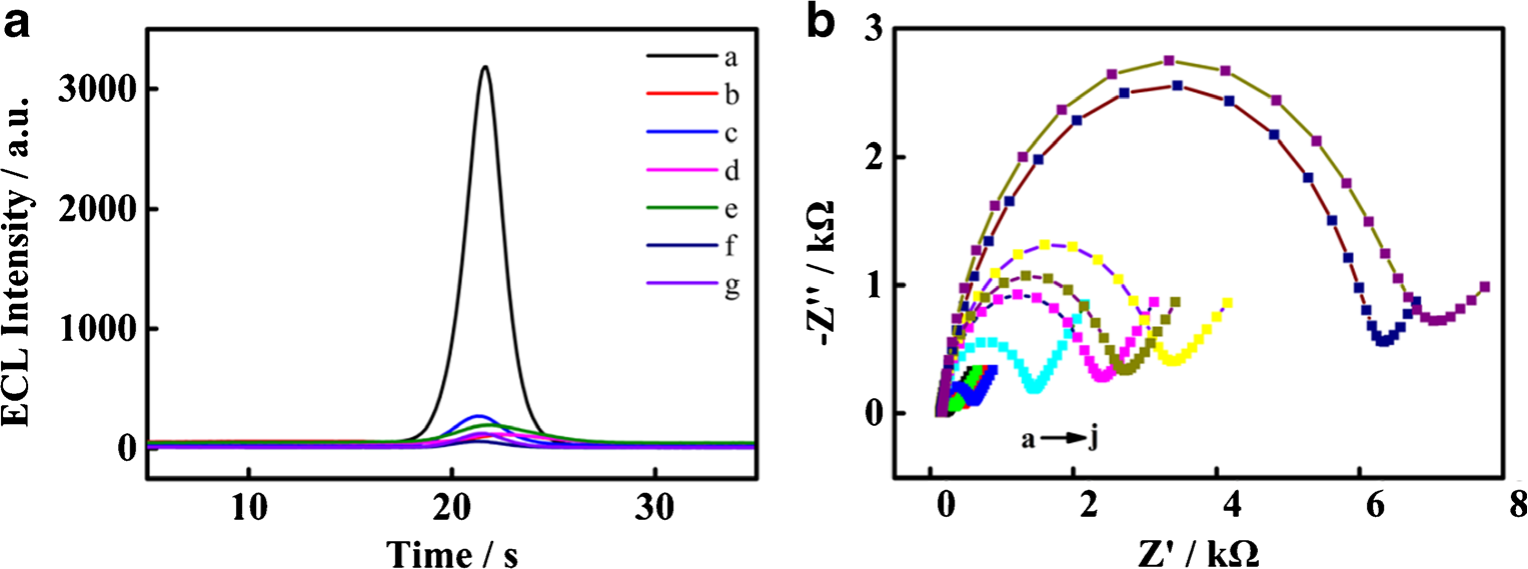
**a** (a) ECL curves of luminol/NAS/Ab2*/CYFRA 21-1/Ab1/MPA/Au (b) in the absence of MPA, (c) Ab1, (d) CYFRA 21-1, (e) initiator, (f) eATRP solution, and (g) luminol. **b** (a) Nyquist plots of Au, (b) MPA/Au, (c) EDC + NHS/MPA/Au, (d) Ab1/EDC + NHS/MPA/Au, (e) BSA1/Ab1/EDC + NHS/MPA/Au, (f) CYFRA 21-1/BSA1/Ab1/EDC + NHS/MPA/Au, (g) BSA2/CYFRA 21-1/BSA1/Ab1/EDC + NHS/MPA/Au, (h) Ab2*/BSA2/CYFRA 21-1/BSA1/Ab1/ EDC + NHS/MPA/Au, (i)eATRP/Ab2*/BSA2/CYFRA 21-1/BSA1/Ab1/EDC + NHS/MPA/Au, and (j) luminol/eATRP/Ab2*/BSA2/CYFRA 21-1/BSA1/Ab1/EDC + NHS/MPA/Au

The modified electrodes under different methods are measured by ECL, verifying the feasibility of the proposed system with regard to the detection of CYFRA 21-1. As in Fig. 1a, no obvious electroluminescent peak can be detected (excluding for a weak background peak) without MPA (curve b), Ab1 (curve c), CYFRA 21-1 (curve d), the initiator (curve e), eATRP solution (curve f), or luminol (curve g). However, for luminol/NAS/Ab2*/CYFRA 21-1/Ab1/MPA/Au (curve a), an obvious electroluminescent signal is visible. The results indicate that the initiator can be introduced for eATRP to amplify the electrochemical signal by using the amide reaction between Ab2 and 2-bromoisobutyric acid. A series of control experiments confirm the feasibility of CYFRA 21-1 detection with eATRP signal amplification.

The concentrations of CYFRA 21-1 are all 1 pg mL^−1^.

### ECL characterization of this biosensor

EIS, in general, monitors the stepwise assembly of the ECL biosensor in the presence of iron ferrocyanides ([Fe(CN)_6_]^3−/4−^, 5.0 mM), with a frequency range of 0.1–100 kHz. The charge-transfer resistance is represented the diameter of the semicircle in the Nyquist diagram [31]. As shown in Fig. 1b, the bare gold electrode indicates the lowest *R*_ct_ because of the rapid electron transfer between the solution and the gold-electrode surface (**~** 240 Ω, Fig. 1b(a)). When MPA is introduced to the electrode surface, *R*_ct_ increases to ~ 461 Ω, because the electrostatic repulsion and hydrophobicity properties of MPA somewhat hinder the electron transfer (Fig. 1b(b)). The results suggest that MPA is successfully immobilized to the electrode surface via the Au–S bond. By contrast, *R*_ct_ decreases after EDC/NHS is added onto the surface, because of the activation of the carboxyl groups on MPA (~ 344 Ω, Fig. 1b(c)). The carboxyl groups with a negative charge at the end of MPA are replaced by succinimide ester, partially eliminating the electrostatic repulsion. However, *R*_ct_ increases consecutively after the successful introduction of Ab1, BSA, CYFRA 21-1, and Ab2 on the electrode (Fig. 1b(d~h)). This is mainly attributed to the fact that proteins reduce the effective active sites in the electron-transfer process. Then, because the polymer chains formed are hydrophobic, *R*_ct_ significantly increases after eATRP, indicating that eATRP is successfully grafted from the electrode surface (~ 6345 Ω, Fig. 1b(i)). When luminol is introduced onto the electrode surface, *R*_ct_ increases to ~ 7177 Ω (Fig. 1b(j)) because of the poor conductivity of luminol, which hinders the charge transfer to some extent. Moreover, those results illustrate that the construction process of this biosensor is successful.

**Fig. 2:**
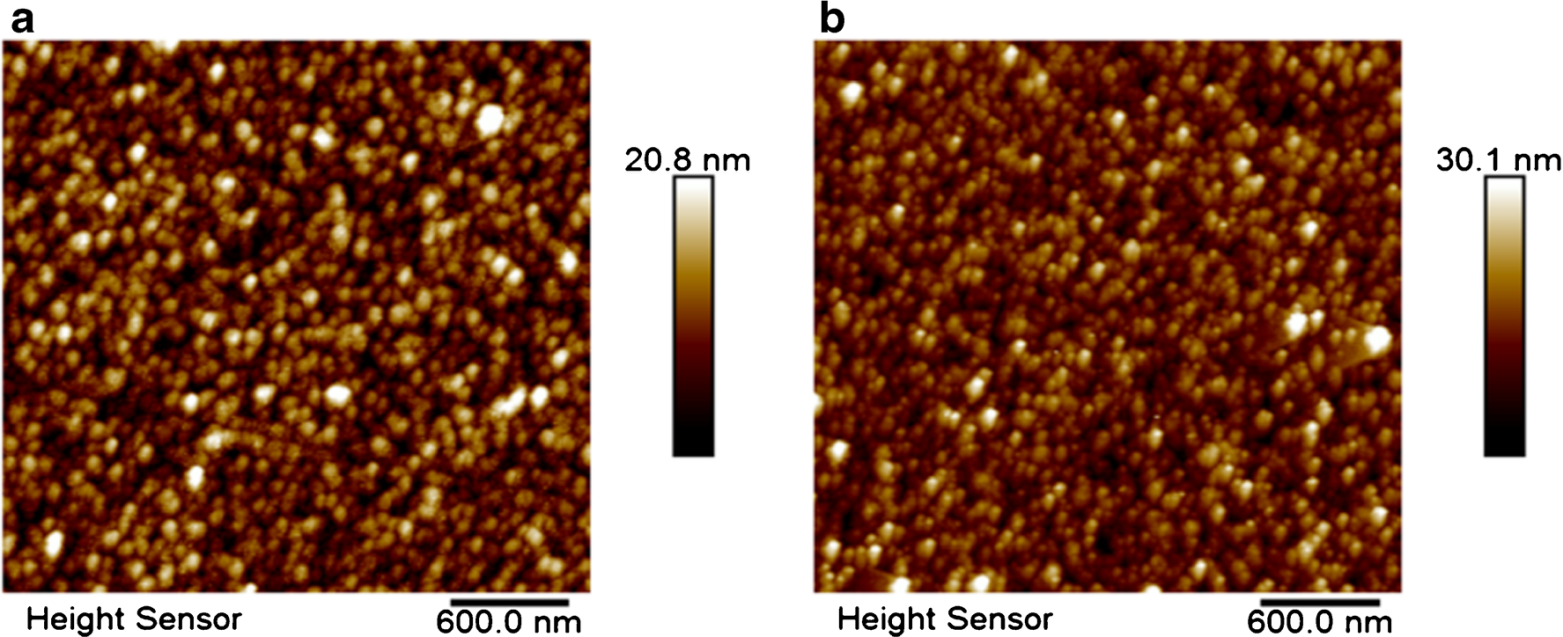
AFM images of the **a** Ab2*/BSA2/CYFRA 21-1/BSA1/Ab1/EDC + NHS/MPA/Au and **b** eATRP/Ab2*/BSA2/CYFRA 21-1/BSA1/Ab1/EDC + NHS/MPA/Au

**Fig. 3:**
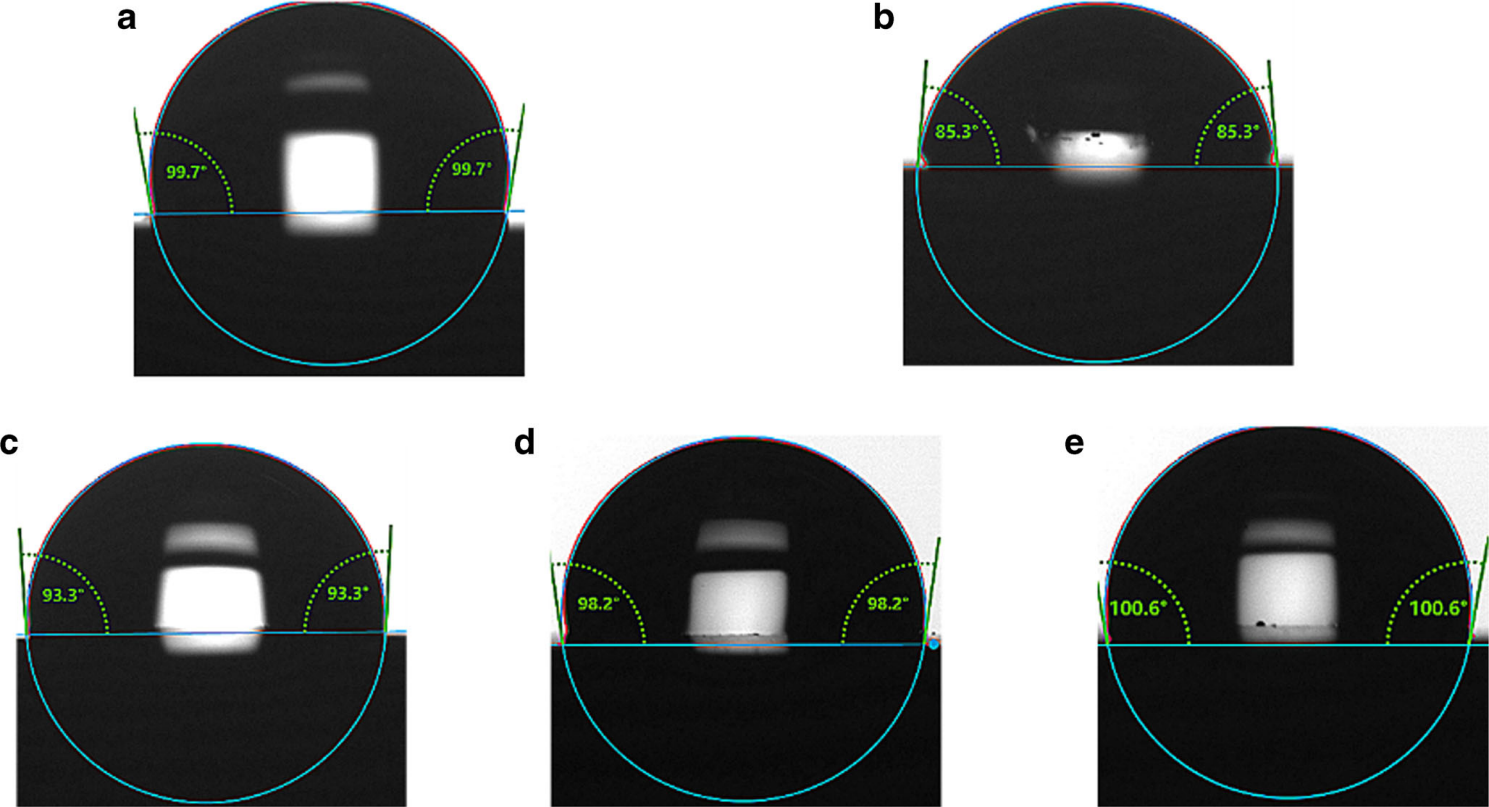
CAM images of **a** Au, **b** MPA/Au, **c** Ab2*/BSA2/CYFRA 21-1/BSA1/Ab1/EDC + NHS/MPA/Au, **d** eATRP/Ab2*/BSA2/CYFRA 21-1/BSA1/Ab1/EDC + NHS/MPA/Au, and **e** luminol/eATRP/Ab2*/BSA2/CYFRA 21-1/BSA1/Ab1/EDC + NHS/MPA/Au

We study the fabrication process of the sensor through the AFM and contact angle. The morphology and surface height are expected to change significantly before and after polymerization. Figure 2 a shows the morphology of Ab2*/BSA2/CYFRA 21-1/BSA1/Ab1/EDC + NHS/MPA/Au, and the surface height increases to 12.7 nm. Subsequently, a high-resolution picture with the surface height of 19.9 nm is gained because of the successful immobilization of considerable NAS by eATRP (Fig. 2b). The wettability and hydrophilicity of the electrode surface depend largely on the chemical properties of the electrode surface. Contact angle measurements (CAMs) can evaluate the success of the biosensor assembly. The CAM results illustrate the CAM evolution as a function of chemical surface modification (Fig. 3). As can be seen, the CAM of the bare gold-electrode surface is recorded at 99.7° (Fig. 3a). Because the carboxyl group on MPA is hydrophilic, the CAM decreases to 85.3° (Fig. 3b) after MPA. After assembling Ab2*, the CAM increases to 93.3° (Fig. 3c) because of the several hydrophobic amino acids, which make the protein hydrophobic. After eATRP, the CAM increases to 98.2° (Fig. 3d), indicating the modification of several hydrophobic NASs on the electrode surface. Subsequently, luminol is introduced on the modified electrode, the CAM increases to 100.6° (Fig. 3e) because of the hydrophobicity of luminol. These experimental results confirm the successful construction of the biosensor based on eATRP.

### Optimization of conditions

In order to enhance the ultrasensitive detection of CYFRA 21-1, it is necessary to optimize the process conditions in the construction of ECL biosensor (see ESM).

### Analytical performance

**Fig. 4:**
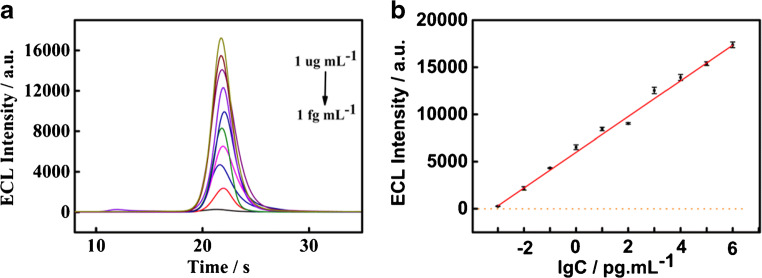
**a** ECL intensity of CYFRA 21-1 at different concentrations from 1 fg mL^−1^ to 1 μg mL^−1^. **b** The calibration plots between ECL signal and logarithm of different CYFRA 21-1 consistencies. The horizontal dashed line represents the ECL level of CYFRA 21-1 at 0 pg mL^−1^

Under the most suitable experimental situations, the response range and limit of detection (LOD) of the ECL biosensor for CYFRA 21-1 detection are quantitatively evaluated. Figure 4 shows the ECL signals at different CYFRA 21-1 consistencies (from 1 fg mL^−1^ to 1 μg mL^−1^) in the incubation solutions. The ECL signal intensity is correlated with the logarithm of CYFRA 21-1 consistencies over 10 orders of magnitude. The linear regression equation is *I* = 1892 log (*C*_CYFRA 21-1_/pg mL^−1^) + 5989 (*R*^2^ = 0.994) and the LOD is 0.8 fg mL^−1^ (*S*/*N* = 3). Here, *S* represents the standard deviation of the control and *N* represents the slope of regression equation. As summarized in Table S1, the amplified sensing strategy has a wider linear range and lower LOD than other methods, because polymerization can introduce many electroactive probes, leading to a significant enhancement of sensitivity.

### Real sample analysis

**Fig. 5:**
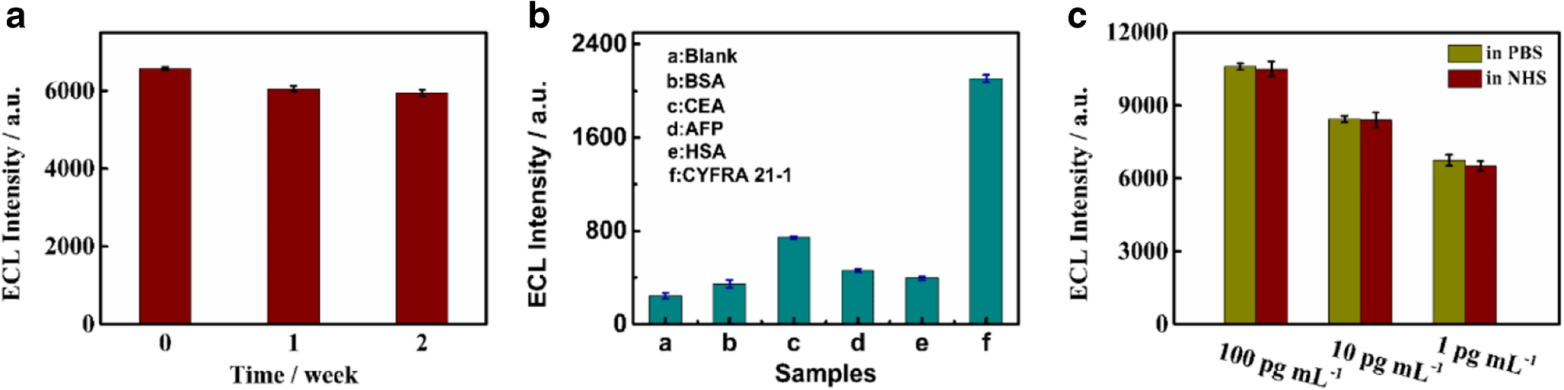
**a** Stability of the ECL-modified electrode with 1 pg mL^−1^ CYFRA 21-1 concentration. **b** The selectivity of the designed ECL biosensor: (a) blank, (b) BSA (1 pg mL^−1^), (c) CEA (1 pg mL^−1^), (d) AFP (1 pg mL^−1^), (e) HAS (1 pg mL^−1^), (f) CYFRA 21-1 (1 pg mL^−1^). **c** Comparison of the ECL signal intensity of the prepared biosensor in 0.1 M PBS (pH 7.4) and 10% human serum samples

The stability of the biosensor is investigated based on the biosensor application. Five electrodes with the same modifications are stored in a refrigerator at 4°, and the water content is saturated. Figure 5 a shows that the ECL signal reaches 90.4% of the initial peak signal after 2 weeks, proposing that the ECL biosensor is a good stable during the storage. To estimate the reproducibility of the developed sensor, the intra-assays (*n* = 5) were executed with 1 pg mL^−1^ CYFRA 21-1. As a result, a relative standard deviation (RSD) value of 0.8% was obtained, which indicated satisfactory reproducibility of the developed sensor.

Moreover, to assess the sensor selectivity, we evaluate the ECL peaks of CYFRA 21-1, BSA, CEA, alpha fetoprotein (AFP), and human serum albumin (HAS) to prove the selectivity of the planned method. The concentrations of all proteins are 10 fg mL^−1^. Figure 5 b shows that the ECL peaks of BSA, CEA, AFP, and HAS are 5.6%, 26.8%, 11.7%, and 8.1% of CYFRA 21-1, respectively, indicating that the biosensor can efficiently distinguish BSA, CEA, AFP, and HSA. According to these results, the planned biosensor is extremely selective for CYFRA 21-1 detection.

To assess the satisfactory practical application of the biosensor, we analyze the ECL peak of CYFRA 21-1 in 10% human serum samples. To prepare a serum sample, CYFRA 21-1 (100 pg mL^−1^, 10 pg mL^−1^, and 1 pg mL^−1^, respectively) is added to 10% of the NHS samples. The conforming ECL peak in 0.1 M PBS contrasts with the ECL peak in the 10% serum sample. Figure 5 c shows that the existing peaks of the 10% human serum samples are 99.0%, 99.3%, and 96.3% of those obtained from 100, 10, and 1 pg mL^−1^ CYFRA 21-1 in 0.1 PBS, respectively. Thus, the ECL biosensor exhibits a decent anti-interference capability for the serum samples, confirming its critical clinical diagnosis possibility.

In order to verify the practicability of the immunosensor in the detection of CYFRA 21-1 in complex biological matrix, seven clinical serum samples from the Third Affiliated Hospital of Henan University of Traditional Chinese Medicine were detected. The results of chemiluminescence analysis of magnetic particles in the hospital and the results of electrochemiluminescence biosensor in this experiment are shown in Table 1. (The sample is diluted serum sample). The relative errors between the results obtained from the two methods ranged from − 2.36 to 5.91%, indicating an acceptable accuracy. This demonstrated that the proposed immunosensor might provide a promising strategy for sensitive determination of CYFRA 21-1 in real serum samples.

**Table 1:** Comparison of detection results from clinical serum samples (*n* = 3)

Sample	Detection results (pg mL^−1^)		Relative error (%)	RSD (%)
	Reference method	Proposed method		
1	0.001385	0.001385	0	2.98
2	0.002750	0.002685	− 2.36	4.45
3	5.583	5.528	− 0.985	1.74
4	0.01026	0.01015	1.1	2.78
5	0.1900	0.1963	2.81	3.30
6	15.093	15.986	5.91	3.66
7	239	227.7	4.32	3.16

## Conclusions

We proposed an ultrasensitive ECL biosensor for detecting CYFRA 21-1 by using the eATRP signal amplification strategy. This was the first combination of ECL and eATRP signal amplification technology. Under the optimized testing conditions, the prepared biosensor allowed an accurate quantification of CYFRA 21-1 in the range of 10 orders of magnitude. Moreover, eATRP supplied various succinimide groups on the side-chain for the local accumulation of luminol. The proposed method provides a promising alternative for CYFRA 21-1 antigen analysis. Furthermore, this proposed sensor can be easily extended to other sensitive biomarkers, such as NSE and CEA.

## Supplementary information

ESM 1 (DOCX 140 kb)
