# Supplementary material for: Electrochemiluminescence immunosensor for cytokeratin fragment antigen 21-1 detection using electrochemically mediated atom transfer radical polymerization
Source: Mikrochim Acta. 2021 Mar 8;188(4):115. doi: 10.1007/s00604-020-04677-x (PMC7940335; doi:10.1007/s00604-020-04677-x)
Supplement: Supplementary file 1 — (DOCX 140 kb) [file 604_2020_4677_MOESM1_ESM.docx]

**Electronic Supplementary Material**

**Electrochemiluminescence immunosensor for cytokeratin fragment antigen 21-1 detection using electrochemically mediated atom transfer radical polymerization**

Lihe Jian^1^**^＆^**, Xiaolan Wang^1,2^**^＆^**, Lulu Hao^1^, Yanju Liu^1^, Huaixia Yang^1^, Xiaoke Zheng^1,2^*,Weisheng Feng^1,2^*

*^1^Pharmacy College, Henan University of Chinese Medicine, Zhengzhou 450046,* *P. R. China*

*^2^The Engineering and Technology Center for Chinese Medicine Development of Henan Province, Zhengzhou 450046, P. R. China*

* Corresponding authors.

Xiaoke Zheng (E-mail: zhengxk.2006@163.com)

Weisheng Feng (E-mail: [fwsh@hactcm.edu.cn](mailto:fwsh@hactcm.edu.cn))

**^＆^**The authors contributed equally to the work.

**Materials**

Cytokeratin fraction 21-1 (CYFRA 21-1), CYFRA 21-1 capture antibody (Ab1), and CYFRA 21-1 detection antibody (Ab2) were acquired from Biodesign (Saco, MA) (https://www.sangon.com/login). N-hydroxysuccinimide (NHS), 2-bromoisobutyric acid, 3-mercaptoproplonlc acid (MPA), bovine serum albumin (BSA), 1-ethyl-3-(3-dimethyllaminopropyl) carbodiimide hydrochloride (EDC), N-acryloxysuccinimide (NAS), and luminol were purchased from Sigma-Aldrich (St. Louis, USA) (https://www.sigmaaldrich.com/united-states.html/). Copper (II) bromide (CuBr_2_) and tris (2-dimethylaminoethyl) amine (Me_6_TREN) were purchased from Lingfeng Chemical Reagent Co., Ltd. (Shanghai, China) (http://zyrfid.cn.cnlinfo.net/gongying/). Human serum was obtained from Shanghai Yi-Ji Industrial Co., Ltd. (Shanghai, China) (https://www.tianyancha.com/company/533919620). PBS buffer (0.1 M, pH = 7.4) was employed as a diluted solution for CYFRA 21-1, Ab1, and Ab2. The eATRP cocktail solution was freshly prepared in the electrolytic cell ([NAS]:[CuBr_2_/Me_6_TREN]:[DMF]:[KPF_6_] =1:1:18:80 v/v). All other reagents were analytical grade or better.

**Apparatus**

The electrochemical and ECL measurements were conducted on an MPI-E multifunctional electrochemical and ECL analytical system (Xi’an Remex Analytical Instrument Ltd. Co., China) (http://www.chinaremex.com/). All ECL measurements were conducted in a 5mL glass cell, comprising a modified gold working electrode, a platinum pair electrode, and an Ag/AgCl (saturated KCl solution) reference electrode. The voltage of the photomultiplier tube (PMT) during detection was set at 1000 V. The cyclic voltammogram (CV) behaviors were analyzed using a CHI832 voltammetric analyzer (Shanghai Chenhua Apparatus Inc, China) (http://www.chinstruments.com/). The electrochemical impedance spectroscopy (EIS) was performed using an Autolabpotentiostat/galvanostat PGSTAT204 (Metrohm, Netherlands) (http://www.metrohm17.com/forestsun-Products-14243537/), with a solution of 0.1 M KNO_3_ containing 5 mM [Fe(CN)_6_]^4−/3−^ (equimolar). Atomic force microscopy (AFM) was conducted using Dimension Icon with a scanning size of 2 μm (Bruker Nano Inc., USA) (<http://www.zhaoyq.com/brand-16.html>).

**Optimization of conditions**

For the planned biosensor, the increase of the number of NAS markers on the electrode surface is the key to improve the luminous intensity. The polymerization time of eATRP is closely related to the length of the grafted polymer chains. Because long-term incubation leads to a longer polymer length on the sensor, the reaction time should be prolonged accordingly. eATRP is a dynamic reversible reaction, which scans are taken every 10 min. Fig. S1A displays the change between the polymerization time and intensity of the light-emitting signal. In the first 30 min, the ECL intensity increases rapidly with the polymerization time. It then increases gradually until reaching a table at 40 min, which might be produced by free radical termination. Hence, 40 min is a suitable eATRP time in our study.

Because of the condensation reaction between the monomer NAS and luminol, the incubation time in the luminol solution is a critical factor affecting the ECL intensity. The ECL intensity of the luminol/NAS/Ab2*/Ag/Ab1/Au electrode increases originally with the development time. The signal reaches the maximum value after a 4 h incubation time. A further prolongation of the incubation time does not lead to the increase of signal, because luminol is almost completely coupled to the available NAS side-chains (Fig. S1B). Therefore, we use an incubation time of 4 h throughout our study.

The intensity of ECL is also related to the concentration of luminol solution. Fig. S1C shows that the effect of the luminol concentration is investigated from 5 to 30 mM. The ECL signal rapidly increases with the luminol consistency, yielding a supreme at 20 mM. This is because as the luminol concentration increases, the coupling reaction between the luminol and NAS side-chain approaches saturation [1]. Consequently, we set the luminol concentration to 20 mM in the following experiments.


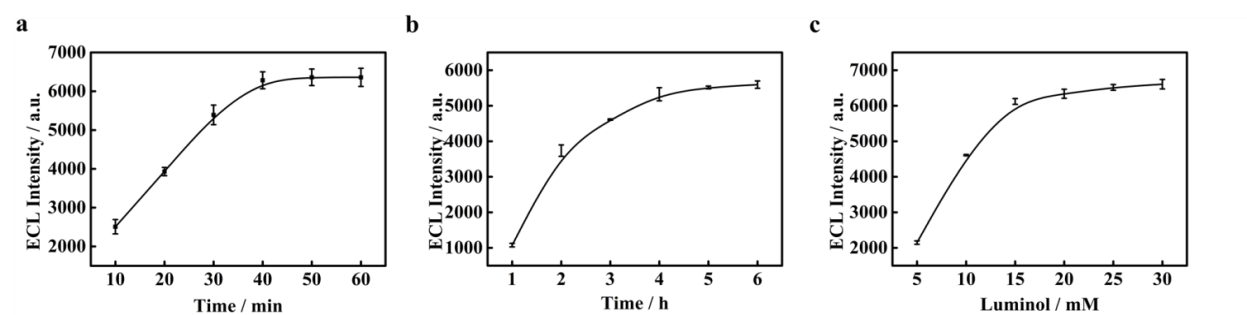


**Fig. S1** The influence ECL intensity **a** of eATRP time **b** of luminol time **c** of luminol concentration. The concentrations of CYFRA 21-1 are all 1 pg mL^−1^.

**Table S1** Comparison of this strategy with other strategies for ultrasensitive protein detection

| Method | Linear range | LOD | Ref |
| --- | --- | --- | --- |
| Electrochemistry | 0.25 to 20 ng mL^−1^ | 1.67 pg mL^−1^ | [2] |
| Electrochemistry | 10^-4^ to 1 ug mL^−1^ | 0.03 ng mL^−1^ | [3] |
| Fluorometry | 10^-2^ to 100 ng mL^−1^ | 8 pg mL^−1^ | [4] |
| ECL | 10^-5^ to 100 ng mL^−1^ | 1.8 fg mL^−1^ | [5] |
| Fluorometry | 0.05 to 2 ng mL^−1^ | 30 pg mL^−1^ | [6] |
| ECL | 10^-5^ to 100 ng mL^−1^ | 1.33 fg mL^−1^ | [7] |
| ECL | 10^-9^ to 1 ug mL^−1^ | 0.9 fg mL^−1^ | This work |

**Reference**s

1. Ariffin EY, Nik Mansor NN, Safitri E, Lee YH, Hassan NI (2019) A Hydrogen Peroxide Biosensor from Horseradish peroxidase Immobilization onto Acrylic Microspheres. Sains Malaysiana 48 (7):1409–1416. doi:10.17576/jsm-2019-4807-09

2. Putnin T, Ngamaroonchote A, Wiriyakun N, Ounnunkad K, Laocharoensuk R (2019) Dually functional polyethylenimine-coated gold nanoparticles: a versatile material for electrode modification and highly sensitive simultaneous determination of four tumor markers. Mikrochim Acta 186 (5):305. doi:10.1007/s00604-019-3370-4

3. Ran G, Wu F, Ni X, Li X, Li X, Liu D, Sun J, Xie C, Yao D, Bai W (2020) A novel label-free electrochemical aptasensor with one-step assembly process for rapid detection of lead (II) ions. Sensors and Actuators B: Chemical 320. doi:10.1016/j.snb.2020.128326

4. Alarfaj NA, El-Tohamy MF, Oraby HF (2020) New Immunosensing-Fluorescence Detection of Tumor Marker Cytokeratin-19 Fragment (CYFRA 21-1) Via Carbon Quantum Dots/Zinc Oxide Nanocomposite. Nanoscale Res Lett 15 (1):12. doi:10.1186/s11671-020-3247-9

5. Liu S, Jia Y, Xue J, Li Y, Wu Z, Ren X, Ma H, Li Y, Wei Q (2020) Bifunctional peptide-biomineralized gold nanoclusters as electrochemiluminescence probe for optimizing sensing interface. Sensors and Actuators B: Chemical 318. doi:10.1016/j.snb.2020.128278

6. Chen M, Ma C, Zhao H, Yan Y (2019) Exonuclease III-assisted fluorometric aptasensor for the carcinoembryonic antigen using graphene oxide and 2-aminopurine. Mikrochim Acta 186 (8):500. doi:10.1007/s00604-019-3621-4

7. Jia Y, Liu S, Du Y, Yang L, Liu X, Liu L, Ren X, Wei Q, Ju H (2020) Intramolecular Coreaction Accelerated Electrochemiluminescence of Polypeptide-Biomineralized Gold Nanoclusters for Targeted Detection of Biomarkers. Anal Chem 92 (13):9179-9187. doi:10.1021/acs.analchem.0c01519
